# The HIV care cascade for adolescents initiated on antiretroviral therapy in a health district of South Africa: a retrospective cohort study

**DOI:** 10.1186/s12879-020-05742-9

**Published:** 2021-01-13

**Authors:** Roxanna Haghighat, Elona Toska, Nontuthuzelo Bungane, Lucie Cluver

**Affiliations:** 1 Department of Social Policy and Intervention, University of Oxford, 32 Wellington Square, Oxford, OX1 3DW UK; 2 Centre for Social Science Research, University of Cape Town, Cape Town, South Africa; 3 Department of Sociology, University of Cape Town, Cape Town, South Africa; 4 Department of Nursing Science, Faculty of Health Sciences, University of Fort Hare, Alice, South Africa; 5 Department of Psychiatry and Mental Health, University of Cape Town, Cape Town, South Africa

**Keywords:** Adolescent, HIV, Antiretroviral therapy, South Africa, Care cascade, Continuum of care

## Abstract

**Background:**

Little evidence exists to comprehensively estimate adolescent viral suppression after initiation on antiretroviral therapy in sub-Saharan Africa. This study examines adolescent progression along the HIV care cascade to viral suppression for adolescents initiated on antiretroviral therapy in South Africa.

**Methods:**

All adolescents ever initiated on antiretroviral therapy (*n*=1080) by 2015 in a health district of the Eastern Cape, South Africa, were interviewed in 2014–2015. Clinical records were extracted from 52 healthcare facilities through January 2018 (including records in multiple facilities). Mortality and loss to follow-up rates were corrected for transfers. Predictors of progression through the HIV care cascade were tested using sequential multivariable logistic regressions. Predicted probabilities for the effects of significant predictors were estimated by sex and mode of infection.

**Results:**

Corrected mortality and loss to follow-up rates were 3.3 and 16.9%, respectively. Among adolescents with clinical records, 92.3% had ≥1 viral load, but only 51.1% of viral loads were from the past 12 months. Adolescents on ART for ≥2 years (AOR 3.42 [95%CI 2.14–5.47], *p*< 0.001) and who experienced decentralised care (AOR 1.39 [95%CI 1.06–1.83], *p*=0.018) were more likely to have a recent viral load. The average effect of decentralised care on recent viral load was greater for female (AOR 2.39 [95%CI 1.29–4.43], *p*=0.006) and sexually infected adolescents (AOR 3.48 [95%CI 1.04–11.65], *p*=0.043). Of the total cohort, 47.5% were recorded as fully virally suppressed at most recent test. Only 23.2% were recorded as fully virally suppressed within the past 12 months. Younger adolescents (AOR 1.39 [95%CI 1.06–1.82], *p*=0.017) and those on ART for ≥2 years (AOR 1.70 [95%CI 1.12–2.58], *p*=0.013) were more likely to be fully viral suppressed.

**Conclusions:**

Viral load recording and viral suppression rates remain low for ART-initiated adolescents in South Africa. Improved outcomes for this population require stronger engagement in care and viral load monitoring.

**Supplementary Information:**

The online version contains supplementary material available at 10.1186/s12879-020-05742-9.

## Background

The expansion of access to antiretroviral therapy (ART) has enabled more children vertically infected with HIV to survive into adolescence [1]. In parallel, the high rate of new HIV infections through sexual transmission among youth has contributed to a growing population of adolescents living with HIV. Of the estimated 2.1 million adolescents living with HIV, 85% reside in sub-Saharan Africa [2, 3]. Concern about the growing population of adolescents requiring lifelong ART has drawn increasing global attention to the need to better monitor this key population and to tailor treatment services [1, 4].

Previous studies have demonstrated that, even on treatment, adolescents exhibit the worst health outcomes compared to all other age groups [2, 5]. In South Africa, which houses the world’s largest ART programme, adolescents have repeatedly demonstrated the lowest rates of retention in care and viral suppression compared to other age groups [6]. The HIV care cascade has been widely used among various patient populations to identify progress at critical stages along the continuum of care from HIV testing to ART initiation and viral suppression [7]. This tool allows for both the monitoring of patients’ health and identification of key gaps in care that can be targeted by interventions in alignment with the UNAIDS 90–90-90 treatment target for 2020 [8, 9].

Although progress along the HIV care cascade has been well-documented globally for adults and adult key populations, less evidence exists for adolescents living with HIV [6, 7, 9]. A recent retrospective analysis of individually linked records from South Africa’s National Health Laboratory Service database estimated that 66% of older adolescents (15–19 years old) had initiated ART by 2016 [10]. While the study provides a robust estimate for the second cascade step of ART initiation, it does not extend analyses to evaluate rates of viral suppression for the third target in the care cascade.

In South Africa, one systematic review of data from youth living with HIV estimated that only 10% were virally suppressed as of 2013, before implementation of universal test and treat in 2016 [6]. However, the review estimated viral suppression using rates reported for patients aged 9–29, well beyond the window of adolescence [11]. Similarly, the 2017 South African National HIV Prevalence, Incidence, Behaviour, and Communication Survey estimated viral suppression for youths aged 15–24, with no discrete data for all adolescents aged 10–19 [12]. This lack of disaggregated data for adolescents limits the applicability of findings for this key population [4]. Furthermore, viral suppression in the survey was calculated by active collection and testing of blood samples, not evaluation of clinical records that would be available to healthcare providers. Research is urgently required to understand the *current* reality of adolescent HIV care progression to the final cascade step of viral suppression in South Africa, with data that reflects contexts beyond well-resourced clinics or urban centres [13].

In this study, we evaluate the progression of ART-initiated adolescents to viral suppression along the HIV care cascade in South Africa, including operational outcomes as well as mortality and retention in care, using data up to 2018 from multiple healthcare facilities within South Africa’s decentralised public healthcare system. In addition, we report associations between stages of the cascade and adolescents’ sociodemographic and treatment-related characteristics.

## Methods

### Participants and procedures

In this longitudinal cohort study, data were analysed from clinical records and interviews with ART-initiated adolescents in a health district of the Eastern Cape, South Africa. The study setting was specifically selected to reflect poverty and healthcare contexts similar to other low-income Southern African contexts and included rural, peri-urban, and urban locations [14]. The province is characterised by high HIV and TB burden, and significant challenges for this province’s healthcare system include limited infrastructure and human resource challenges [15]. Adolescent participants were recruited from March 2014 through September 2015, as previously described [16]. All healthcare facilities within the health district that provided ART to at least five adolescents were included in the study (*n*=52, including eight hospital wards, five community health centres, and 39 primary care clinics). At each healthcare facility, patient registers were reviewed to identify all adolescents aged 10–19 years who had ever initiated ART, including those who had defaulted in care or been lost to follow up (LTFU). Adolescents identified through clinical records were traced to >180 communities, approached for study participation, and, if consenting, interviewed at their preferred location.

### Data collection

#### Clinical record review

At each of the 52 healthcare facilities, routine clinical records were searched for every study participant, across both paper-based and electronic records. This intensive data collection approach enables the extraction of participants’ records from all included facilities where they may have received care, including undocumented silent transfers to a new facility. Data were extracted in two rounds using a standardized form, covering records from 2014 to 2015 and 2016–2017 respectively, and included plasma viral load, CD4 cell count, and WHO staging.

#### Adolescent interviews

At two study waves (0 and 18 months follow-up), adolescents completed tablet-based surveys in their preferred language, with the support of research assistants trained in working with South African adolescents. Surveys assessed adolescents’ experiences at home, in their communities, and in healthcare settings, and questions were developed to be understandable and non-stigmatising through extensive stakeholder consultation, including with South African adolescents living with HIV [16, 17]. For the present analyses, self-reported data were only used to determine participants’ sociodemographic and treatment-related characteristics. Further study information, including study protocol, is available at www.mzantsiwakho.org.za.

### Measures

Operational outcomes were evaluated in the HIV care cascade between ART initiation and viral suppression and included the following: availability of any clinical records, having at least one viral load recorded within clinical records, and having a viral load recorded within the past 12 months (backdated from the date of extraction). Finally, viral suppression was measured at two thresholds (HIV-1 RNA <1000 copies/mL and full viral suppression designated at HIV-1 RNA <50 copies/mL) [18]. Viral suppression was determined using the most recent viral load available across records, including in multiple facilities. Mortality was determined using both clinical records and community tracing through May 2018. LTFU was defined as recorded in participants’ most recent clinical records, adjusted for mortality and silent transfers into care at new facilities. Silent transfers were identified when a patient re-entered care in a new facility without an official notification to the former facility of care. Participants without any available clinical records were assumed to be LTFU.

In total, seven covariates were included in analyses [1]: Age (younger adolescents aged 10–14 years vs. older adolescents aged 15–19 years) [2]; sex [3]; residential location at baseline (urban vs. rural) [4]; sexual vs. vertical mode of infection (determined following existing sub-Saharan African paediatric cohorts: age of ART initiation cut-off [≤10 years] [19], validated and updated with a detailed algorithm that considered other strong evidence (i.e. self-reported sexual history and parental death) [20] [5]; time on ART (≥2 vs. <2 years, measured from ART initiation to date of viral load) [6]; experiencing decentralised care (either exclusively at primary care clinics or experienced down-referral from a higher- to lower-level facility); and [7] mortality. For analyses of viral suppression, age at most recent viral load was binarized and used as the age covariate (10–14 vs. 15–19 years). For other outcomes, binarized age at study enrolment was used (10–14 vs. 15–19 years). Mortality was included as a covariate only for analyses of operational outcomes (having a clinical record and having at least one viral load in clinical records).

### Statistical analysis

First, we summarised sociodemographic and treatment-related characteristics of the cohort using descriptive statistics. Second, we calculated the relevant proportion of participants who had reached each step of the HIV care cascade, including operational outcomes. The proportion of participants with clinical records was measured out of the total study cohort, and the proportion of participants with available viral loads was calculated relative to participants with available clinical records. The proportions of participants with viral loads recorded in the past 12 months and viral suppression were calculated relative to the number of participants with at least one recorded viral load.

Third, we tested associations between each step of the HIV care cascade and socio-demographic and treatment-related predictors, using a sequential multivariable logistic regression approach following Hosmer and Lemeshow [21]. All covariates were simultaneously included in the first model and then removed sequentially: the second and third models retained only variables significant at *p *<0.10 and *p *<0.05, respectively. For each cascade outcome, the final model controlled for test multiplicity, using the Benjamini-Hochberg step-up procedure to correct for false discovery rate [22]. For all models, correlation matrices indicated no multicollinearity between variables. All possible two- and three-way interactive effects between covariates significant in the final model were also tested and corrected using the Benjamini-Hochberg procedure and the Wald test for significance. Fourth, predicted probabilities for covariates significant in the final model were estimated by sex and mode of infection [23]. Multivariable logistic regressions and marginal probability models were performed using SPSS version 23 (IBM Corp, Armonk, NY) and STATA/SE 15.1 (StataCorp, College Station, TX), respectively.

### Ethical approval

Ethical approval for this study was given by the University of Oxford (SSD/CUREC2/12–21), University of Cape Town (CSSR 2013/4; 2017/3), Eastern Cape Departments of Health and Basic Education, and ethical review boards of all participating healthcare facilities. At both study waves, adolescent participants and their caregivers provided voluntary, informed, and written consent for participation, including interviews and access to adolescents’ clinical records. Initial review of patient registers in healthcare facilities was only used to identify eligible participants, and further access to clinical records was obtained only after adolescents consented to study participation. In cases of low literacy among adolescents or caregivers, all information and consent procedures were read aloud in the participant’s preferred language. There were no financial incentives for study participation, but all participants received a certificate of participation, snacks, and a small gift pack including pencils and soap. Adolescents who refused to participate were still given snacks. To minimise risk of stigma, all potentially publicly available materials, such as participation certificates and general information pamphlets, referred to the study as one about general adolescent health in South Africa.

## Results

A total of 1080 ART-initiated adolescents were recruited into the study. Years of ART initiation ranged from 2000 to 2017, providing 6921 person-years of study follow-up across participants. Median age at study enrolment was 13 years (IQR: 11–16 years), and 55.3% of participants were female (Table 1). Most participants were living in urban locations (76.7%) and vertically infected (74.8%). In the total cohort, mortality and corrected LTFU rates (including those without clinical records) were 3.3% (*n*=36) and 16.9% (*n*=183), respectively. Overall, 37.9% of all facility-designated LTFU patients (*n*=95) were found to be misclassified: 7.4% (*n*=7) due to unrecorded mortality, and 30.5% (*n*=29) from re-entry into care after silent transfer to a new facility.

**Table 1:** Baseline demographic and clinical characteristics of study cohort

	Number of participants, % (***n***=1080)
**Sex**	
Female	597 (55.3%)
Male	483 (44.7%)
**Location**	
Urban	828 (76.7%)
Rural	252 (23.3%)
**Age**	
Age at study enrolment (yrs, median (IQR))	13 (11–16)
Age at ART initiation (yrs, median (IQR))*	9 (6–12)
**Mode of infection**	
Vertically infected (n,%)	808 (74.8%)
Sexually infected (n,%)	272 (25.2%)
**Year of ART initiation**	
2000–2009	436 (40.4%)
2010–2013	373 (34.5%)
2014–2017	114 (10.6%)
Data not available	157 (14.5%)
**Baseline viral load**	
<50 copies/mL	99 (9.2%)
51–999 copies/mL	401 (37.1%)
≥1000 copies/mL	378 (35.0%)
Data not available	202 (18.7%)
**Baseline CD4 count**	
<200 cells/mm^3^	205 (19.0%)
200–349 cells/mm^3^	192 (17.8%)
≥350 cells/mm^3^	515 (47.7%)
Data not available	169 (15.6%)
**Baseline WHO Clinical Stage**	
Stage I	283 (26.2%)
Stage II	167 (15.5%)
Stage III	212 (19.6%)
Stage IV	29 (2.7%)
Data not available	388 (35.9%)
**Follow-up time**	
Study follow-up time since ART initiation (yrs, median (IQR))*	7.2 (4.7–9.8)

ART: Antiretroviral therapy; IQR: Interquartile Range; LTFU: Lost to follow-up

* Median values calculated among participants with available clinical records

**Fig. 1:**
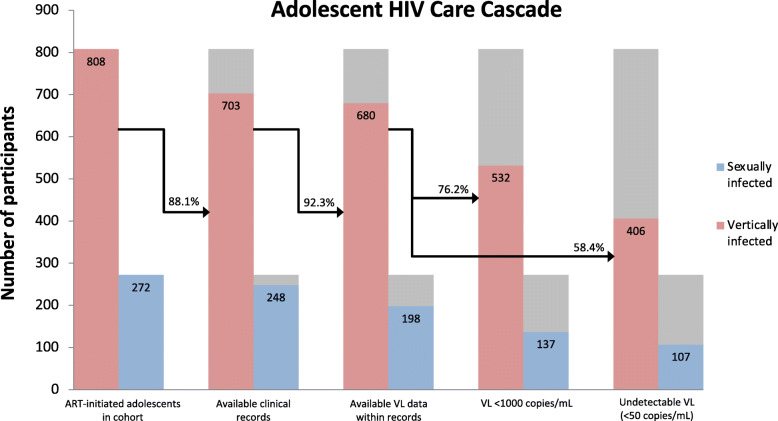
Adolescent HIV care cascade by mode of infection

Of the total adolescent cohort, 88.1% (*n*=951) had at least one clinical record available (Fig. 1). Attending multiple care facilities was common, with 29.8% (*n*=283) of participants having records from at least two facilities. In total, 51.3% (*n*=488) experienced decentralised care.

Among those with clinical records, median age at ART initiation was 9 years (IQR: 6–12 years), and median follow-up time since ART initiation was 7.2 years (IQR: 4.7–9.8 years). At baseline on ART, the majority of adolescents were viraemic, with 72.1% reporting detectable viral loads (≥50 copies/mL) (Table 1). At baseline, at least 19.0% were severely immunocompromised (CD4 <200 cells/mm [3]), and at least 22.3% were either moderately or severely symptomatic (WHO Clinical Stage III/IV).

Among those with clinical records, a further 92.3% (*n*=878) had at least one viral load recorded within their files (Fig. 1). Of those with any viral load data, 75.4% (*n*=662) and 51.1% (*n*=449) had their most recent viral load recorded in the past 24 and 12 months, respectively. Median age at most recent viral load was 14 years (IQR: 12–17 years). Approximately equal proportions of adolescents had their most recent viral load recorded at a hospital (39.0%, *n*=342) and a primary care clinic (39.7%, *n*=349), with 21.3% (*n*=187) recorded at a community health centre. At most recent viral load, 74.1% (*n*=651) were on first-line ART with an NNRTI backbone, and 19.2% (*n*=169) were on second-line ART with a PI backbone. A further 6.6% (*n*=58) were on an NRTI-only or unspecified ARV regimen.

At most recent viral load, 76.2% (*n*=669) of those with viral load data were virally suppressed at <1000 copies/mL, and 58.4% (*n*=513) were fully virally suppressed at <50 copies/mL (Fig. 1). This rate corresponds to 47.5% full viral suppression at most recent viral load for the total adolescent cohort. Only 23.2% (*n*=251) of the total cohort demonstrated full viral suppression within the past 12 months.

In the final multivariable model, male (AOR 1.55 [95%CI 1.05–2.28], *p*=0.026) and sexually infected adolescents (AOR 1.71 [95%CI 1.06–2.74], *p*=0.028) were more likely to have an available clinical record (Table 2). However, sexual infection (AOR 0.28 [95%CI 0.15–0.52], *p *<0.001) and older age at study enrolment (AOR 0.32 [95%CI 0.16–0.62], *p*=0.001) were associated with lower likelihood of having at least one recorded viral load. Male (AOR 2.36 [95%CI 1.28–4.37], *p*=0.006) and rural-living adolescents (AOR 2.13 [95%CI 1.07–4.27], *p*=0.033) were more likely to have a viral load available in clinical records (Table 2).

**Table 2:** Final models for associations between HIV care cascade outcomes and socio-demographic and treatment-related characteristics

	Available clinical record (*n*=951) AOR (95% CI); ***p*** value	Available VL data within records (*n*=878) AOR (95% CI); ***p*** value	VL recorded in past 12 months (*n*=449) AOR (95% CI); ***p*** value	Most recent VL <1000 copies/mL (*n*=669) AOR (95% CI); ***p*** value	Most recent VL <50 copies/mL (*n*=513) AOR (95% CI); ***p*** value
**Mortality**	–	–			
**Rural living**	–	2.13 (1.07–4.27); 0.033	–	0.66 (0.46–0.93); 0.019	–
**Sex (male)**	1.55 (1.05–2.28); 0.026	2.36 (1.28–4.37); 0.006	–	–	–
**Age at study enrolment (≥15 years)**	–	0.32 (0.16–0.62); 0.001	0.82 (0.62–1.10); 0.181		
**Sexually infected**	1.71 (1.06–2.74); 0.028	0.28 (0.15–0.52); <0.0001	–	–	–
**Decentralised care**		–	1.39 (1.06–1.83); 0.018	–	–
**Time on ART (≥2 years)**			3.42 (2.14–5.47); <0.0001	1.72 (1.09–2.72); 0.020	1.70 (1.12–2.58); 0.013
**Age at most recent VL (≥15 years)**				0.54 (0.39–0.75); <0.0001	0.72 (0.55–0.94); 0.017

*AOR* Adjusted odds ratio, *ART* Antiretroviral therapy, *CI* 95% Confidence interval, *VL* Viral load

Note: Adjusted odds ratios are only indicated for variables remaining in the final model for each outcome. Empty boxes indicate variables that were not applicable to analyses. Boxes filled with a dash indicate variables not included in the final model because they had failed to reach significance in prior models

Longer time on ART (AOR 3.42 [95%CI 2.14–5.47], *p *<0.001) and experiencing decentralised care (AOR 1.39 [95%CI 1.06–1.83], *p*=0.018) were associated with having a viral load recorded in the past 12 months (Table 2). Longer time on ART was also associated with viral suppression at <1000 copies/mL (AOR 1.72 [95%CI 1.09–2.72], *p*=0.020). However, adolescents who were older at most recent viral load (AOR 0.54 [95%CI 0.39–0.75], *p *<0.001) and rural-residing (AOR 0.66 [95%CI 0.46–0.93], *p*=0.019) were less likely to be virally suppressed at this level. Similarly, adolescents on ART for longer were more likely to be fully virally suppressed at <50 copies/mL (AOR 1.70 [95%CI 1.12–2.58], *p*=0.013), but adolescents who were older at most recent viral load were less likely to be fully virally suppressed (AOR 0.72 [95%CI 0.55–0.94], *p*=0.017). Full results for all model stages for each cascade outcome are provided in Additional Files 1 and 2. No significant three-way interactions were found between variables significant in the final models for any outcome.

**Fig. 2:**
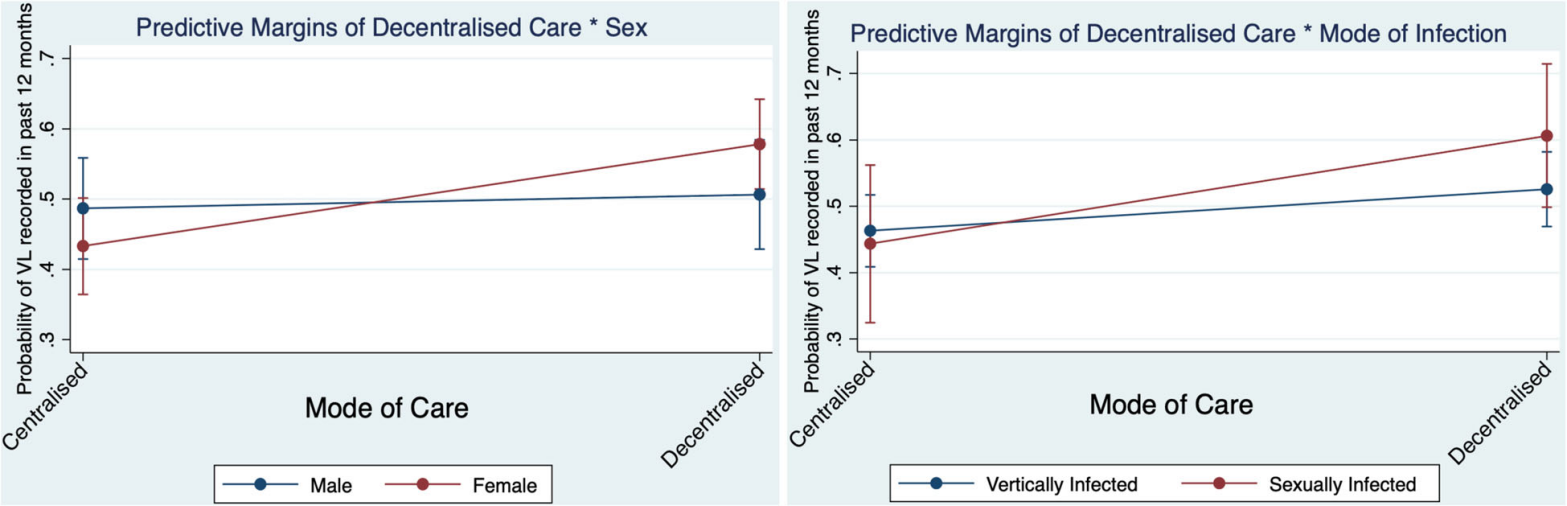
Predicted probabilities of decentralised care by sex (left) and mode of infection (right) on recent viral load

In the marginal probability model, the average effect of decentralised HIV care on likelihood of having a viral load in the past 12 months was greater for females than males (AOR 2.39 [95%CI 1.29–4.43], Wald χ^2^=7.60, *p*=0.006), and greater for sexually infected adolescents than their vertically infected counterparts (AOR 3.48 [95%CI 1.04–11.65], Wald χ^2^=4.10, *p*=0.043) (Fig. 2).

## Discussion

This study provides a comprehensive estimate of the current reality of adolescent HIV care and progression to viral suppression through the HIV care cascade in South Africa’s public healthcare system, including operational outcomes. These results highlight not only the low rate of past-year viral suppression among adolescents but also the importance of timely viral load monitoring. To our knowledge, this is the first study to evaluate both the timing and rate of viral suppression for a large cohort of adolescents across facilities in a sub-Saharan African public healthcare setting [4, 24].

By tracing participants in the community and across all 52 public facilities, this HIV care cascade adjusted for under-reported mortality and silent transfers in estimates of LTFU. At 16.9%, this study suggests that the true rate of LTFU is higher than previously reported for vertically infected adolescents in sub-Saharan Africa. This difference may result from this study’s inclusion of adolescents sexually infected with HIV, a sub-population previously found to be at higher risk for LTFU [5, 24].

This study found that roughly 38% of adolescents designated as LTFU by facilities were misclassified due to under-reported mortality and silent transfers. The importance of including silent transfers for accurately assessing patient outcomes has been noted elsewhere, particularly in settings like South Africa without universal unique patient identifiers [25]. The high rate of silent transfer may reflect adolescents’ residential mobility; adolescents’ actively self-specialising care services across multiple sites; or the effect of decentralising HIV care, through which patients’ care is distributed across healthcare levels [25, 26]. Therefore, facility-based approaches to HIV care and treatment in South Africa must account for adolescents’ mobility across sites.

Findings suggest that South African adolescent progression to the final “90” of viral suppression in the UNAIDS 90–90-90 treatment goal remains low, at 47.5% for ART-initiated adolescents aged 10–19 years. Therefore, this study extends evidence on the adolescent HIV care cascade in South Africa, beyond ART initiation rates previously reported by Maskew and colleagues to the next step of viral suppression, linked across healthcare facilities [10]. This low rate of viral suppression in clinical records corroborates previously published findings on ART adherence from this adolescent cohort, which identified 49% self-reported past-week ART adherence among adolescents at the study’s 18-month follow-up interview [14]. Hence, findings suggest that critical challenges persist in adolescent attainment of consistent ART adherence and viral suppression after treatment initiation.

Further, this study investigated the critical implementation question of the frequency of viral load testing. This operational outcome reflects the extent to which adolescent HIV care in South Africa’s decentralised health system actually adheres to national guidelines. National guidelines recommend at least annual viral load testing for all adolescents, even in the most decentralised community-based care models [11]. We found that only half of adolescents with available viral loads had their most recent viral load tested in the past 12 months. This rate of adolescents’ viral load testing is far below the provincial estimate of 80% of all ART-initiated patients having a viral load within the past 12 months [27]. Although adolescents’ viral load availability may be high, these results suggest that adolescents’ viral loads are often out-dated. Because viral load monitoring is the primary approach for South African healthcare providers to determine patients’ clinical care needs, the lack of recent viral load data available in routine clinical records may significantly reduce their ability to make appropriate care decisions [11].

Only 23.2% of all adolescents were documented to be virally suppressed in the past year. This low rate of past-year viral suppression potentially provides a more meaningful representation of the current state of adolescent HIV care in South Africa. As younger adolescents age and become sexually active and have children, routine viral load monitoring and suppression become even more crucial for prevention of onwards transmission to partners and children [1]. While longer time on ART and receiving decentralised care were associated with having a viral load reported in the past 12 months, the protective effect of decentralised care was stronger for females and for sexually infected adolescents. These sub-populations may be more likely to engage with decentralised care through access to sexual and reproductive health services at primary care clinics.

This study has several limitations. Because study eligibility was limited to ART-initiated adolescents, findings are not generalisable to rates of viral suppression for adolescents living with HIV who have not been tested or initiated on ART, in the first two steps of the UNAIDS 90–90-90 target and care cascade. However, this study’s inclusion of adolescents who were LTFU or disengaged from care minimised the risk of sample selection bias. Because this retrospective cohort study analysed routine clinical records, it is only able to present estimates based on data that would be available to healthcare providers in facilities. Rates of viral suppression presented in this study represent the proportion of adolescents known and documented to have achieved viral suppression. Although adolescents without any electronic or paper-based viral load records could potentially be virally suppressed, this information was not available. In line with previous studies, these adolescents were presumed to be disengaged from care and thus likely to have unsuppressed viral loads [6, 28].

Additionally, the present study is unable to systematically identify precise reasons for out-dated or missing viral loads for each participant. Potential factors include both provider-related challenges (inconsistent record-keeping systems resulting in missed scheduling, invalid test results, or lost test results), and patient-related challenges (true disengagement from care, being unwilling or unable to queue for blood tests, or having a proxy pick up medications and report on their health) [29, 30]. Potential transfers to facilities beyond the health district or into private care were not captured.

However, this study has several significant methodological strengths. Notably, participant tracing in the community allowed for correction of facilities’ mortality records. Tracing individual patients across 52 healthcare facilities enabled a more accurate evaluation of adolescents’ health by including unrecorded patient-initiated silent transfers to new facilities [24, 25]. This improved estimates for not only LTFU but also most recent viral loads. Analysis of clinical records through December 2017 also allowed for evaluation of adolescent viral load monitoring and HIV care outcomes in the era of universal test and treat. These outcomes reflect the current reality of how ART-initiated adolescents are receiving follow-up care and responding to treatment within a healthcare system responsible for a larger patient population than even before. Additionally, through linking adolescents’ self-reported information to clinical records, this cohort study was able to evaluate the effects of individual-level predictors on progression along the HIV care cascade, which has not been possible in previous analyses of records from national databases [10, 24]. Finally, by evaluating adolescents’ clinical records across 52 public facilities in the Eastern Cape, findings potentially represent outcomes for adolescents in similar settings across South Africa and sub-Saharan Africa—beyond findings from highly resourced and externally supported clinics.

Further longitudinal analysis of outcomes is required to investigate the cyclical nature of engagement with HIV care, in order to identify when and which adolescents disengage from the HIV care cascade [31]. Future research within this adolescent cohort should also examine self-reported clinical care experiences as predictors of HIV care outcomes. Identifying reasons for gaps in the operational outcomes from this HIV care cascade and strategies to improve timely viral load monitoring for adolescents is critical for effective programmatic and policy changes.

## Conclusions

This study documents progression of ART-initiated adolescents across the HIV care cascade to viral suppression in a sub-Saharan African public healthcare setting, accounting for mortality and silent transfers. Adolescents living with HIV in the Eastern Cape, South Africa, demonstrate low rates of viral suppression, and viral load monitoring practices fall well below national guidelines. The effectiveness of UNAIDS 90–90-90 goals for achieving HIV treatment success requires not only viral suppression but also routine, up-to-date viral load monitoring and recording.

## Supplementary Information

**Additional file 1 Table S1.** Full results from sequential multivariable logistic regression analyses of associations between baseline sociodemographic and treatment-related variables and operational HIV care outcomes.

**Additional file 2 Table S2.** Full results from sequential multivariable logistic regression analyses of associations between baseline sociodemographic and treatment-related variables and viral load outcomes.

## Data Availability

The datasets generated and analyzed for the current study are not publicly available due to ethical and confidentiality constraints but can be available from the corresponding author on reasonable request within the purview of prior ethical approvals.

## Abbreviations

AOR: Adjusted odds ratio
ART: Antiretroviral therapy
ARV: Antiretroviral
CI: Confidence interval
HIV: Human immunodeficiency virus
IQR: Interquartile range
LTFU: Loss to follow-up
NNRTI: Non-nucleoside reverse transcriptase inhibitor
NRTI: Nucleoside reverse transcriptase inhibitor
PI: Protease inhibitor
TB: Tuberculosis
UNAIDS: Joint United Nations Programme on HIV/AIDS
VL: Viral load
WHO: World Health Organization
