# Supplementary material for: The HIV care cascade for adolescents initiated on antiretroviral therapy in a health district of South Africa: a retrospective cohort study
Source: BMC Infect Dis. 2021 Jan 13;21:60. doi: 10.1186/s12879-020-05742-9 (PMC7805141; doi:10.1186/s12879-020-05742-9)
Supplement: Supplementary file 1 — Additional file 1 Table S1. Full results from sequential multivariable logistic regression analyses of associations between baseline sociodemographic and treatment-related variables and operational HIV care outcomes. [file 12879_2020_5742_MOESM1_ESM.docx]

**Table S1: Full results from sequential multivariable logistic regression analysis testing association between baseline sociodemographic and treatment-related variables and operational outcomes in the HIV care cascade (having an available clinical record and having at least one viral load recorded within clinical records).** Goodness of model fit was assessed via Hosmer and Lemeshow test.

|  | **Available clinical record (n=951/1080)** | | | **Available VL data within clinical record (n=878/951)** | | |
| --- | --- | --- | --- | --- | --- | --- |
|  | **AOR** | **Lower CI** | **Upper CI** | **AOR** | **Lower CI** | **Upper CI** |
| **Step 1** |  |  |  |  |  |  |
| Mortality | 0.84 | 0.32 | 2.23 | 0.60 | 0.18 | 1.99 |
| Rural living | 0.97 | 0.63 | 1.50 | 2.30* | 1.14 | 4.63 |
| Sex (male) | 1.55* | 1.05 | 2.28 | 2.31** | 1.24 | 4.29 |
| Age at study enrollment (≥15 years) | 0.96 | 0.62 | 1.50 | 0.35** | 0.18 | 0.70 |
| Sexually infected | 1.74* | 1.02 | 2.98 | 0.29** | 0.15 | 0.53 |
| Decentralised care | - | - | - | 0.59^†^ | 0.34 | 1.03 |
| **Step 2** |  |  |  |  |  |  |
| Mortality | - | - | - | - | - | - |
| Rural living | - | - | - | 2.32* | 1.15 | 4.67 |
| Sex (male) | 1.55* | 1.05 | 2.28 | 2.30** | 1.24 | 4.27 |
| Age at study enrollment (≥15 years) | - | - | - | 0.34** | 0.17 | 0.67 |
| Sexually infected | 1.71* | 1.06 | 2.74 | 0.29** | 0.16 | 0.54 |
| Decentralised care | - | - | - | 0.59^†^ | 0.34 | 1.02 |
| **Step 3** |  |  |  |  |  |  |
| Mortality | - | - | - | - | - | - |
| Rural living | - | - | - | 2.13* | 1.07 | 4.27 |
| Sex (male) | - | - | - | 2.36** | 1.28 | 4.37 |
| Age at study enrollment (≥15 years) | - | - | - | 0.32** | 0.16 | 0.62 |
| Sexually infected | - | - | - | 0.28** | 0.15 | 0.52 |
| Decentralised care | - | - | - | - | - | - |
| **Final model fit** (*Χ*^2^(df), *p*) | *Χ*^2^ (2) = 0.48, *p* = 0.789 | | | *Χ*^2^ (6) =6.55, *p* = 0.365 | | |

AOR: Adjusted odds ratio; ART: Antiretroviral therapy; CI: 95% Confidence interval; VL: Viral load

^†^*p*<0.1; * *p*<0.05 ; ** *p*<0.01
