# Supplementary material for: The HIV care cascade for adolescents initiated on antiretroviral therapy in a health district of South Africa: a retrospective cohort study
Source: BMC Infect Dis. 2021 Jan 13;21:60. doi: 10.1186/s12879-020-05742-9 (PMC7805141; doi:10.1186/s12879-020-05742-9)
Supplement: Supplementary file 2 — Additional file 2 Table S2. Full results from sequential multivariable logistic regression analyses of associations between baseline sociodemographic and treatment-related variables and viral load outcomes. [file 12879_2020_5742_MOESM2_ESM.docx]

**Table S2: Full results from sequential multivariable logistic regression analysis testing association between baseline sociodemographic and treatment-related variables and viral load outcomes in the HIV care cascade (having a viral load recorded in the past 12 months, most recent viral load <1000 copies/mL, and most recent viral load <50 copies/mL).** Goodness of model fit was assessed via Hosmer and Lemeshow test.

|  | **VL recorded in past 12 months (n=449/878)** | | | **Most recent VL <1000 copies/mL (n=669/878)** | | | **Most recent VL <50 copies/mL (n=513/878)** | | |
| --- | --- | --- | --- | --- | --- | --- | --- | --- | --- |
|  | **AOR** | **Lower CI** | **Upper CI** | **AOR** | **Lower CI** | **Upper CI** | **AOR** | **Lower CI** | **Upper CI** |
| **Step 1** | | | | | | | | | |
| Mortality | - | - | - | - | - | - | - | - | - |
| Rural living | 0.93 | 0.67 | 1.29 | 0.65* | 0.45 | 0.94 | 0.77 | 0.56 | 1.06 |
| Sex (male) | 1.02 | 0.77 | 1.35 | 0.84 | 0.61 | 1.17 | 0.90 | 0.68 | 1.18 |
| Age at study enrollment (≥15 years) | 0.72^†^ | 0.52 | 1.00 | - | - | - | - | - | - |
| Sexually infected | 1.39^†^ | 0.95 | 2.03 | 0.83 | 0.56 | 1.24 | 1.00 | 0.70 | 1.43 |
| Decentralised care | 1.40* | 1.06 | 1.85 | 0.99 | 0.72 | 1.38 | 0.80 | 0.60 | 1.05 |
| Time on ART (≥2 years) | 3.63** | 2.25 | 5.85 | 1.68* | 1.05 | 2.69 | 1.75* | 1.14 | 2.68 |
| Age at most recent VL (≥15 years) | - | - | - | 0.56** | 0.40 | 0.79 | 0.71* | 0.53 | 0.96 |
| **Step 2** | | | | | | | | | |
| Mortality | - | - | - | - | - | - | - | - | - |
| Rural living | - | - | - | 0.66* | 0.46 | 0.93 | - | - | - |
| Sex (male) | - | - | - | - | - | - | - | - | - |
| Age at study enrollment (≥15 years) | 0.72* | 0.52 | 1.00 | - | - | - | - | - | - |
| Sexually infected | 1.39^†^ | 0.95 | 2.02 | - | - | - | - | - | - |
| Decentralised care | 1.38* | 1.05 | 1.81 | - | - | - | - | - | - |
| Time on ART (≥2 years) | 3.61** | 2.24 | 5.81 | 1.72* | 1.09 | 2.72 | 1.70* | 1.12 | 2.58 |
| Age at most recent VL (≥15 years) | - | - | - | 0.54** | 0.39 | 0.75 | 0.72* | 0.55 | 0.94 |
| **Step 3** | | | | | | | | | |
| Mortality | - | - | - | - | - | - | - | - | - |
| Rural living | - | - | - | - | - | - | - | - | - |
| Sex (male) | - | - | - | - | - | - | - | - | - |
| Age at study enrollment (≥15 years) | 0.82 | 0.62 | 1.10 | - | - | - | - | - | - |
| Sexually infected | - | - | - | - | - | - | - | - | - |
| Decentralised care | 1.39* | 1.06 | 1.83 | - | - | - | - | - | - |
| Time on ART (≥2 years) | 3.42** | 2.14 | 5.48 |  |  |  |  |  |  |
| Age at most recent VL (≥15 years) | - | - | - | - | - | - | - | - | - |
| **Final model fit** (*Χ*^2^(df), *p*) | *Χ*^2^ (3) = 0.45, *p* = 0.929 | | | *Χ*^2^ (5) = 4.04, *p* = 0.543 | | | *Χ*^2^ (1) = 0.06, *p* = 0.815 | | |

AOR: Adjusted odds ratio; ART: Antiretroviral therapy; CI: 95% Confidence interval; VL: Viral load

^†^*p*<0.1; * *p*<0.05 ; ** *p*<0.01
